# Supplementary material for: Low Molecular Weight Fucoidan Prevents Radiation-Induced Fibrosis and Secondary Tumors in a Zebrafish Model
Source: Cancers (Basel). 2020 Jun 18;12(6):1608. doi: 10.3390/cancers12061608 (PMC7353073; doi:10.3390/cancers12061608)
Supplement: Supplementary file 1 [file cancers-12-01608-s001.pdf]

cancers

MDPI

Article

Low Molecular Weight Fucoidan Prevents Radiation-Induced Fibrosis and Secondary Tumors in A Zebrafish Model

Szu-Yuan Wu [1,2,3,4,5], Wan-Yu Yang [6], Chun-Chia Cheng [6,7], Ming-Chen Hsiao [8], Shin-Lin Tsai [6], Hua-Kuo Lin [6], Kuan-Hao Lin [6] and Chiou-Hwa Yuh [6,9,10,11,*]

1 Department of Food Nutrition and Health Biotechnology, College of Medical and Health Science, Asia University, Taichung 42354, Taiwan; szuyuanwu5399@gmail.com
2 Division of Radiation Oncology, Department of Medicine, Lo-Hsu Medical Foundation, Lotung Poh-Ai Hospital, Yilan 265, Taiwan
3 Big Data Center, Lo-Hsu Medical Foundation, Lotung Poh-Ai Hospital, Yilan 265, Taiwan
4 Department of Healthcare Administration, College of Medical and Health Science, Asia University, Taichung 41354, Taiwan
5 School of Dentistry, College of Oral Medicine, Taipei Medical University, Taipei 110, Taiwan
6 Institute of Molecular and Genomic Medicine, National Health Research Institutes, Zhunan, Miaoli 35053, Taiwan; cs081011@nhri.edu.tw (W.-Y.Y.); cccheng.biocompare@gmail.com (C.-C.C.); aenny716@gmail.com (S.-L.T); hklin66@gmail.com (H.-K.L.); khlin@nhri.edu.tw (K.-H.L.)
7 Radiation Biology Research Center, Institute for Radiological Research, Chang Gung University/Chang Gung Memorial Hospital at Linkou, Taoyuan 33302, Taiwan
8 Research and Development Center, Hi-Q Marine Biotech International Ltd. Songshan District, Taipei 10561, Taiwan; ming.hsiao@hiqbio.com
9 Institute of Bioinformatics and Structural Biology, National Tsing-Hua University, Hsinchu 30013, Taiwan
10 Department of Biological Science & Technology, National Chiao Tung University, Hsinchu 30010, Taiwan
11 Program in Environmental and Occupational Medicine, Kaohsiung Medical University, Kaohsiung 80708, Taiwan
* Correspondence: chyuh@nhri.edu.tw; Tel.: +886-37-246-166 (ext. 3538); Fax: +886-37-586-459

Supplementary Materials:

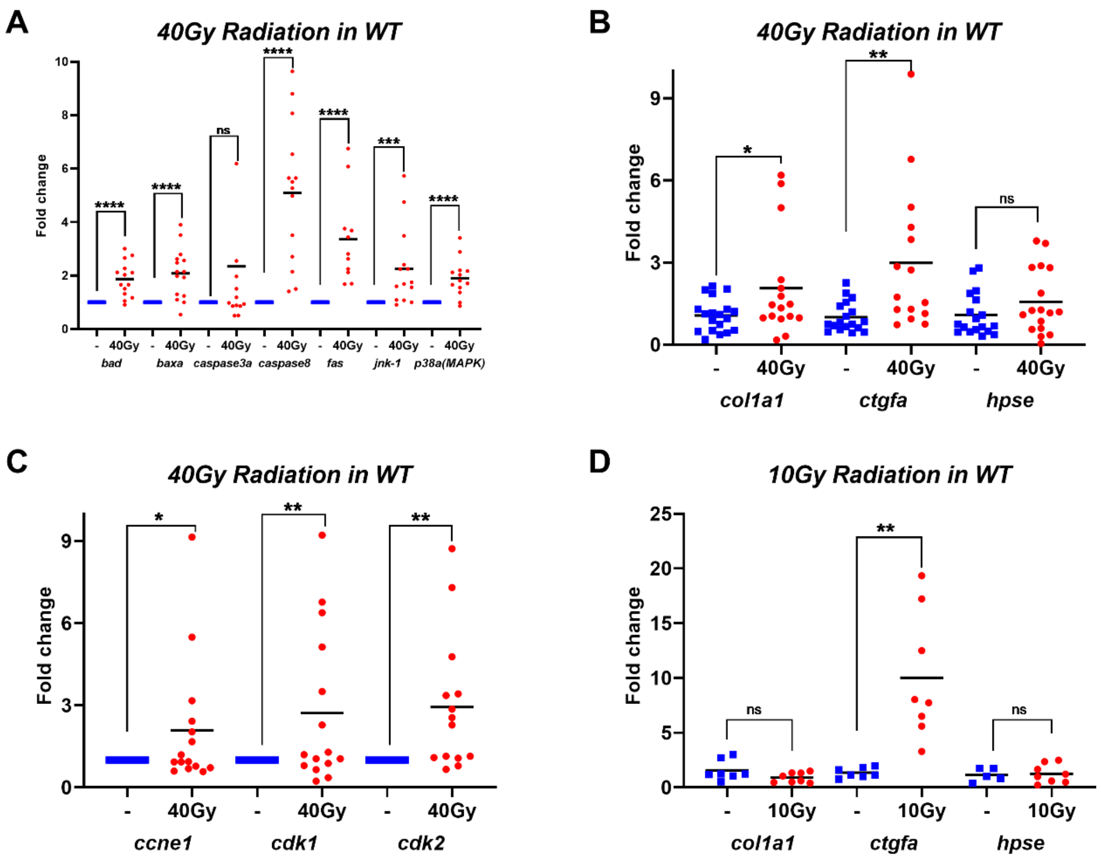

Figure S1. Expression level of apoptosis, fibrosis, and cell proliferation-related genes in adult wild-type zebrafish without or with radiation. (A) The expression of bcl2 associated agonist of cell death (*bad*), bcl2-associated X protein a (*baxa*), apoptosis-related cysteine peptidase 3a (*caspase 3a*), apoptosis-related cysteine peptidase 8 (*caspase 8*), apoptosis antigen 1 (*fas*), c-Jun N-terminal kinase 1 (*jnk-1*), mitogen-activated protein kinase 8a (*p38(MAPK)*) in wild-type fish 40 Gy irradiation (40 Gy) or without irradiation (-). **(B)** The expression of collagen, type I, alpha 1a (*col1a1*), connective tissue growth factor a (*ctgfa*), heparanase (*hpse*) in wild-type fish 40 Gy irradiation (40 Gy) or without irradiation (-). **(C)** The expression of cyclin E1 (*ccne1*), cyclin-dependent kinase 1 (*cdk1*) and cyclin-dependent kinase 2 (*cdk2*) in wild-type fish 40 Gy irradiation (40 Gy) or without irradiation (-). **(D)** The expression of *col1a1*, *ctgfa* and *hpse* in wild-type fish 10 Gy irradiation (10 Gy) or without irradiation (-). The data are presented as dot plots with a horizontal line for the mean and are repeated in triplicate. The statistical significance was calculated using Student's t-test (*$P < 0.05$, **$P < 0.01$, ***$P < 0.001$).

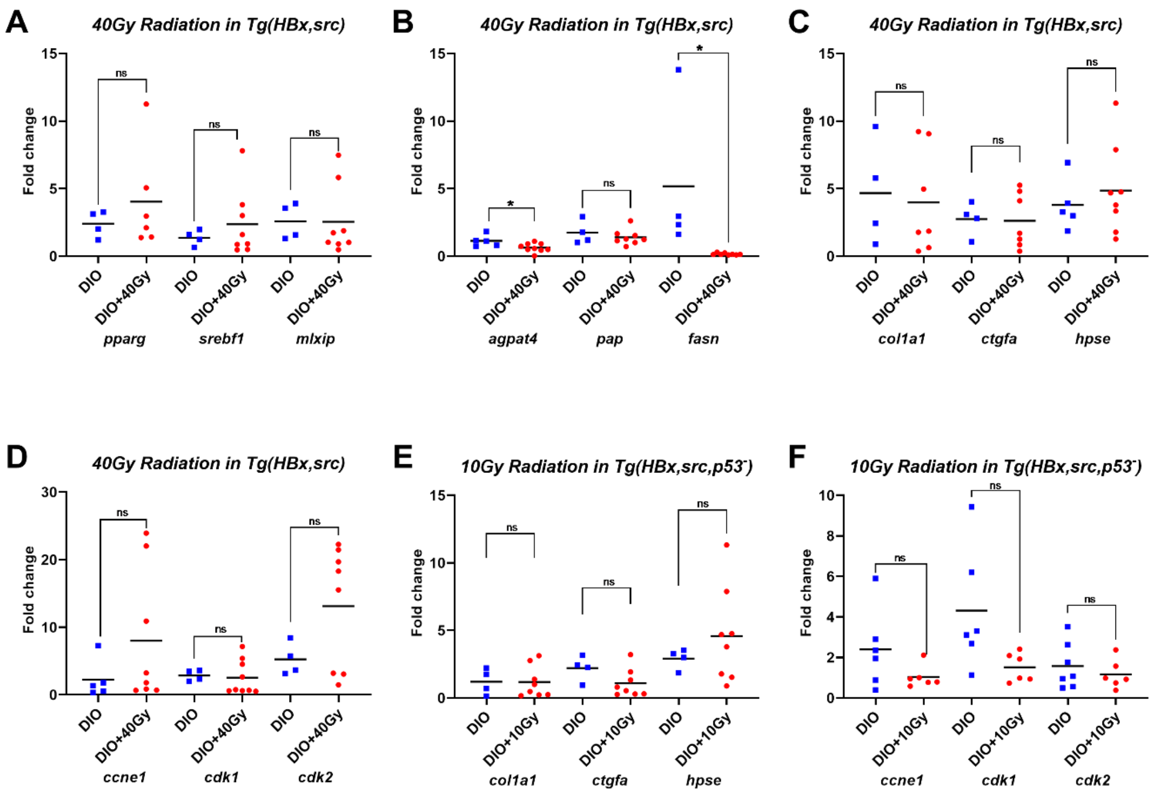

Figure S2. Expression levels of lipogenic factor and lipogenesis enzyme genes in adult transgenic zebrafish without or with radiation. (A) The expression of lipogenic factors (*pparg, srebp1,* and *mlxlp*) in HBx,src transgenic fish with diet-induced obesity (DIO) or DIO plus 40 Gy irradiation (DIO+40Gy). **(B)** The expression of lipogenesis enzyme 1-acylglycerol-3-phosphate O-acyltransferase 4 (lysophosphatidic acid acyltransferase, delta) (*agpat4*) , phospholipid phosphatase 1a (plpp1a), transcript variant X1, mRNA (*pap*), and fatty acid synthase (*fasn*) in HBx,src transgenic fish with diet-induced obesity (DIO) or DIO plus 40 Gy irradiation (DIO+40Gy). **(C)**. The expression of collagen, type I, alpha 1a (*col1a1*), connective tissue growth factor a (*ctgfa*), heparanase (*hpse*) in HBx,src transgenic fish with diet-induced obesity (DIO) or DIO plus 40 Gy irradiation (DIO+40Gy). **(D)**. The expression of cyclin E1 (*ccne1*), cyclin-dependent kinase 1 (*cdk1*) and cyclin-dependent kinase 2 (*cdk2*) in HBx,src transgenic fish with diet-induced obesity (DIO) or DIO plus 40 Gy irradiation (DIO+40Gy). **(E)**. The expression of *col1a1*, *ctgfa*, and *hpse* in HBx,src,p53- transgenic fish with diet-induced obesity (DIO) or DIO plus 10 Gy irradiation (DIO+10Gy). **(F)**. The expression of *ccne1*, *cdk1* and *cdk2* in HBx,src,p53- transgenic fish with diet-induced obesity (DIO) or DIO plus 10 Gy irradiation (DIO+10Gy).

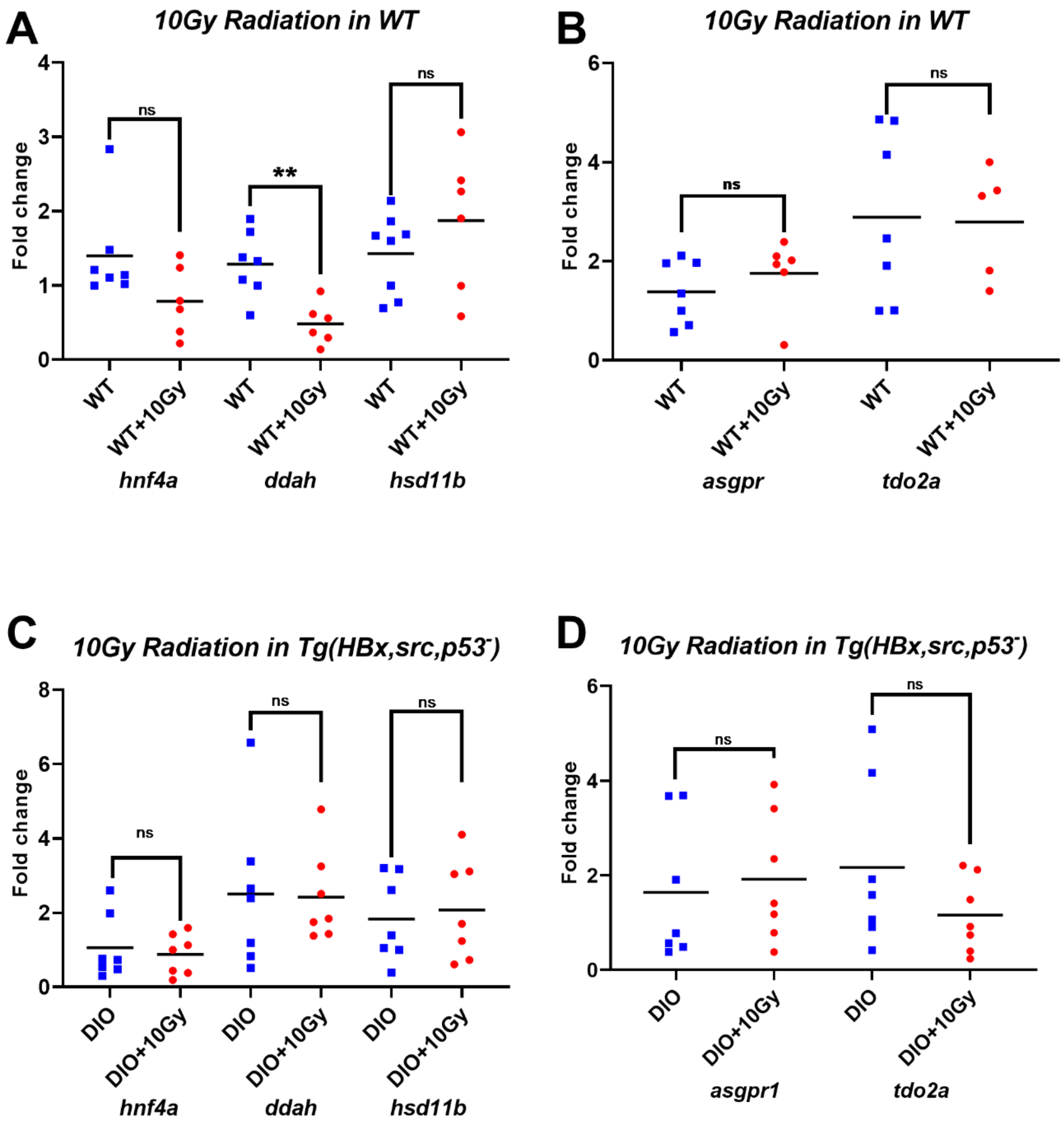

Figure S3. The expression of *hnf4a, ddah1, 11b-hsd1, asgpr1*, and *hnf4a* downstream target genes: *tdo2a*, in zebrafish after irradiation. **(A)** Gene expression profiles of *hnf4a, ddah1, 11b-hsd1* and **(B)** *zhi(asgpr1), tdo2a* in wild-type fish 10 Gy irradiation (WT+10 Gy) or without irradiation (WT). **(C)** Gene expression profiles of *hnf4a, ddah1, 11b-hsd1* and **(D)** *zhi(asgpr1), tdo2a* in HBx,src,p53- transgenic fish with diet-induced obesity (DIO) or DIO plus 10 Gy irradiation (DIO+10Gy). Statistical significance was calculated by t-test (*p ≤ 0.05, **p ≤ 0.01, ***p ≤ 0.001).

TABLE S1. PRIMER SEQUENCES

Gene name	Primer name	Sequence (5′ to 3′)	Accession number	Size
**lipogenic enzyme**				
fasn	Q-*fasn*-F	ATCTGTTCCTGTTCGATGGC	XM_682295	250
	Q-*fasn*-R	AGCATATCTCGGCTGACGTT		
pap	Q-*pap*-F	CAGTTCTTCCTGATTGCTGC	XM_692415	250
	Q-*pap*-R	TCCTCAAAGCTTAGTTCGGG		
agpat4	Q- *agpat4*-F	TTGGCGAAAAAGGAACTGTC	NM_212992	250
	Q- *agpat4*-R	GGTGGTACTTGAGTTTGGGG		
**cell cycle/division-related genes**				
ccne1	Q-*ccne1*-F	TCCCGACACAGGTTACACAA	NM_130995.1	201
	Q-*ccne1*-R	TTGTCTTTTCCGAGCAGGTT		
cdk1	Q-*cdk1*-F	CTCTGGGGACCCCTAACAAT	NM_212564.2	200
	Q-*cdk1*-R	CGGATGTGTCATTGCTTGTC		
cdk2	Q-*cdk2*-F	CAGCTCTTCCGGATATTTCG	NM_213406.1	199
	Q-*cdk2*-R	CCGAGATCCTCTTGTTTGGA		
**fibrosis marker genes**				
col1a1a	Q-*col1a1a*-F	TATTGGTGGTCAGCGTGGTA	NM_199214.1	199
	Q-*col1a1a*-R	TCCTGGAGTACCCTCACGAC		
ctgfa	Q-*ctgfa*-F	TGTGTGTTTGGTGGAATGGT	NM_001015041.2	198
	Q-*ctgfa*-R	GGAGTCACACACCCACTCCT		
hpse	Q-*hpse*-F	GCTCTGGTTTGGAGCTCATC	NM_001045005.1	203
	Q-*hpse*-R	GAAATCCCGACCAAGTTGAA		
**cell death/stress-related genes**				
badb	Q-*badb*-F	AAGCCTGGATAAACACAAGA	NM_131579.1	111
	Q-*badb*-R	ATGAGGATCTTCTGCAACTC		
baxa	Q-*baxa*-F	TGCAAAGCATGTTAAACAAC	NM_131562.2	195
	Q-*baxa*-R	TCCCTGATCCAGTTAATGAC		
caspase3a	Q-*casp3*a-F	GCATCATCATCAACAACAAG	NM_131877.2	133
	Q-*casp3a*-R	GGCAACTGTTGTTAAAACCT		
caspase8	Q-*casp8*-F	TCCAATCGAAGCTGATTTCC	NM_131510.2	166
	Q-*casp8*-R	GCATCTGCTTGTAGCCCTTC		
fas	Q-*fas*-F	CACAACACAGTGTGCCATGA	XM_685355.3	160
	Q-*fas*-R	GGGAGGTGAGGGCTTAAATC		
jnk-1	Q-*jnk-1*-F	CTGATTCAGAACACAGCAAA	XM_017358756.2	162
	Q-*jnk-1*-R	ACTGTGTGTTCCCTCTCATC		
p38a(MAPK)	Q-*p38a*-F	CTCGACACACACAGATGATGAG	NM_131722.1	202
	Q-*p38a*-R	GCTAGGCATCCTGCTTATTA		
**GeneTitan genes**				
hnf4a	Q-13279312-F	AGCCGTGTGGCTGTAAGAAT	NM_194368.1	250
	Q-13279312-R	GTAGTGTCGGCAACAGCAGA		
ddah1	Q-13273615-F	GATCCTGGCCAACACCTTTA	NM_213276.1	229
	Q-13273615-R	CGGCAGGTTCATGTACACAC		
11b-hsd1	Q-13092949-F	TTGCTGATTGCTGTCCTCAC	XM_009298924.3	216
	Q-13092949-R	CTTAGCGCCCAGTTTCTCAC		
tdo2a	*tdo2a*-F	GTTCCTTTCCAGCTGCTGAC	NM_001102616.2	195
	tdo2a-R	CGTGGCCAGGTTAAACAGAT		

asgpr1(zhi)	*asgpr1(zhi)*-F	TGGAAAACTGCAGAAAGCAA	XM_005167373.3	242
	asgpr1(zhi)-R	CAATCCTCACCATTCACACG		
**internal control**				
actin	Q-*actin* -F	CTCCATCATGAAGTGCGACGT	NM_131031.1	180
	Q-*actin* -R	CAGACGGAGTATTTGCGCTCA		
